# Levels of selected metals in leaves of *Cannabis sativa* L. cultivated in Ethiopia

**DOI:** 10.1186/s40064-015-1145-x

**Published:** 2015-07-16

**Authors:** Agalu Zerihun, Bhagwan Singh Chandravanshi, Ayalew Debebe, Bewketu Mehari

**Affiliations:** Forensic Investigation Directorate, Ethiopian Federal Police Commission, Addis Ababa, Ethiopia; Department of Chemistry, College of Natural Sciences, Addis Ababa University, P.O. Box 1176, Addis Ababa, Ethiopia

**Keywords:** Cannabis, *Cannabis sativa* L., Trace metals, Flame atomic absorption spectrometry, Ethiopia

## Abstract

**Background:**

*Cannabis sativa* L. is one of the illicit drug bearing plants. Cannabis products are the most widely trafficked drugs worldwide. The highest levels of cannabis production in the world take place in the African continent. A small volume of cannabis is produced in rural areas of Ethiopia, of which a small portion is exported to neighboring countries and the majority is consumed at home. The literature survey revealed that there is no report on the metal contents in cannabis cultivated in Ethiopia. The main objective of this study is to determine the level of selected metals in leaves of *Cannabis sativa* L. cultivated in Ethiopia.

**Methods:**

*Cannabis sativa* L. samples were collected from Metema (Amhara Region), Mekelle (Tigray Region), Sheshemene (Oromia Region) and Butajira (South Nations Nationality and Peoples (SNNP) Region) of Ethiopia. After proper sample pretreatment, the volumes of reagents used, digestion temperature and digestion time were optimized and using the optimized conditions the levels of metals were determined by flame atomic absorption spectrometry.

**Results:**

The accuracy of the optimized procedure was evaluated by analyzing the digest of the spiked samples with standard solution and the percentage recoveries varied from 88 to 103%. The levels of metals determined (µg/g dry weight) were in the ranges Ca (657–1,511), Zn (321–380), Ni (124–172), Cu (122–176), Cd (3–10), Pb (8–10), and Cr (4–8). Zn was with the highest concentration among trace metals.

**Conclusion:**

A statistical analysis of variance (ANOVA) at 95% confidence level indicated that there is a significant difference in the levels of all the metals between the four sample means except Pb. The results indicate that the content of Pb and Cd exceeds the permissible amount for medicinal plants which form the raw materials for the finished products set by World Health Organization (WHO).

## Background

Cannabis (*Cannabis sativa* L.) is an annual herbaceous plant. It is a dioecious plant (Suurkuusk 2010). In many countries, cannabis is cultivated as a narcotic substance or a source of narcotic substances like hashish and hashish oil. Now a day cannabis is cultivated on the large areas with the mild and tropical climate for the cannabis oil and fibre (United Nations on Drugs and Crimes 2009). The highest levels of cannabis production in the world take place in the African continent. The crude drug can be obtained from leaves, flowers, seeds and stem of cannabis. The female plant yield more drug than the male. It can be smoked in cigarettes or pipes and can be snuffed or added to food (Eboh and Thomas 2005).

Cannabis products are the most widely trafficked drugs worldwide. Practically all countries in the world are affected by cannabis trafficking (United Nations on Drugs and Crimes 2009). Ethiopia does not play a major role in the production, trafficking or consumption of illicit narcotics or precursor chemicals associated with the drug trade. A small amount of cannabis is produced in rural areas of Ethiopia, of which a small portion is exported, primarily to the neighboring countries; the majority is consumed at home, but absolute quantities in both cases are moderate (INCSR 2006).

The main areas of cannabis cultivation in Ethiopia are Alemaya district of eastern Hararghe, Shebedino district of Sidamo, Sheshemene district and other areas in Oromia region, and Debre Berhan district in Amhara region. Now, Sheshemene is famous for the quality of its cannabis produced and its involvement in the commercial cannabis trade in Ethiopia and surrounding countries (Sensi 2014).

The chemical composition of cannabis varies with the type, age and part (flower, root, leaf, fiber, etc.) of cannabis plant as well as with the type of preparation. While ∆^9^-tetrahydrocannabinol is responsible for psychoactive properties of cannabis some of the other components modulate its activity.

*Cannabis sativa* L. is known for millennia for its therapeutic properties and as a recreational psychoactive drug. The adverse health effects of cannabis are a source of contention in debates about policies towards the drug. Cannabis can adversely affect some users, especially adolescents who initiate use early and young adults who become regular users (Hall 2009).

Herbal medicines are likely to be contaminated with heavy metals. In trace amounts some heavy metals are essential for the human body however they may be toxic if present in a higher concentration. They have the ability to bioaccumulate and disrupt functions of vital organs and glands in the human body such as brain, kidney and liver (Dzomba et al. 2012).

The high concentration of heavy metals in soils is reflected by higher concentrations of metals in plants, and consequently in animal and human bodies (Buszewski et al. 2000). The uptake mechanism is selective, plants preferentially acquiring some metals over others (Angelova et al. 2004).

Recently some studies have been carried out on levels of essential and non-essential metals in a psychoactive khat leaves (Atlabachew et al. 2010) and some medicinal plants cultivated in Ethiopia (Derbie and Chandravanshi 2011; Gebre and Chandravanshi 2012; Aregahegn et al. 2013; Mekebo and Chandravanshi 2014; Endalamaw and Chandravanshi 2015; Dubale et al. 2015; Wagesho and Chandravanshi 2015).

Levels of selected heavy metals have been determined in cannabis in some countries. In Pakistan heavy metals (Cr, Pb, Cu, Cd, Ni and Zn) were determined in *Cannabis Sativa* L. and the soil of the area from where the plant was collected using AAS. The plant parts including root, stem and leaves were found to have the quantity of heavy metals corresponding to their contents in the soil (Khan et al. 2008).

Another research conducted on the distribution of heavy metals content in cannabis leaf and seed were carried out using AAS in Nigeria. The results showed that As, Cd., Cr, Fe, Ni, Pb and Hg levels in cannabis leaf exceeded those of the cannabis seed. Therefore cannabis leaves seem to be more dangerous to health than the seeds (Eboh and Thomas 2005).

The levels of some heavy metals (Pb, Ni, Cd and Cr) in eight different medicinal plants samples including *Cannabis sativa* L. along with soils were collected from two different locations from salt range of Punjab, Pakistan in order to evaluate those vital metals involved in human health implications. The findings suggest that the use of these plant species for the management of diseases will not cause heavy metal toxicity (Khan et al. 2013).

The levels of selected heavy metals (Fe, Mn, Cu, Zn and Pb) and macronutrients (Na, Ca, Mg, K and P) in eight useful herbal plants including *Cannabis sativa* L. in Pakistan were determined by AAS. The levels of heavy metals determined in the analyzed samples were found below the maximum allowable limit. The analyzed samples were good source of important macro elements (Ghani et al. 2012).

The literature survey revealed that there is no report on the metal contents in cannabis cultivated in Ethiopia. The main objective of this study is to determine the level of selected metals in leaves of *Cannabis sativa* L. cultivated in Ethiopia. The specific objectives are: (1) to develop an optimum working procedure for digestion of leaves of *Cannabis sativa* L. samples to determine selected metals by flame atomic absorption spectrophotometry (FAAS), (2) to determine the concentrations of Ca, Cr, Ni, Cu, Zn, Cd and Pb in leaves of *Cannabis sativa* L., (3) to compare the levels of the identified metals in leaves of *Cannabis sativa* L. from different regions of Ethiopia, and (4) to compare the levels of the identified metals in leaves of *Cannabis sativa* L. in Ethiopia and with that of the data in the literature.

## Methods

### Equipments

Polyethylene bags were used during sample collection, sample drying and preserving the ground and homogenized samples. Electronic blending device, Moulinex (Ecully Cedex, France) was used for grinding and homogenizing the sample. Electronic series balance, WL 3000, Adam Equipment Co. Ltd. (Milton Keynes, UK), with precision of ±0.0001 g was used for weighing samples. 100 mL round bottom flasks fitted with reflux condensers were used in Kjeldahl, Fisher Scientific UK Ltd (Leicestershire, UK) apparatus hot plate to digest the dried and powdered cannabis samples. Borosilicate volumetric flasks (25, 50 and 100 mL) were used during dilution and storage of samples and preparation of metals standard solutions. 1, 2, 5 and 10 mL pipettes, Pyrex (St. Louis, MO, USA) were used for measuring the solutions. Refrigerator, Hitachi (Tokyo, Japan) was used for the preservation of digested samples before AAS analysis was made. Micropipettes (10–100 µL and 100–1,000 µL), Dragonmed (Shangai, China) were used for measuring different volumes of metal standard solutions for spiking. Flame atomic absorption spectrophotometer, Analytik Jena ZEEnit 700P (Jena, Germany) equipped with deuterium arc back ground connectors and hollow cathode lamps with air-acetylene flame was used for the determination of the analyte metals (Ca, Cr, Ni, Cu, Zn, Cd and Pb) in the samples.

### Reagents and chemicals

The chemicals and reagents used in the study were HNO_3_ 69.5% (Scharlau Chemie S.A., European Union, Spain), HClO_4_ 70% (Reaserch-Lab Fine Chem Industries, Mumbai, India), H_2_SO_4_ 98% (Research-Lab Fine Chem Industries, Mumbai, India), La(NO_3_)_3_.6H_2_O 98% pure (BDH Chemicals Ltd, Poole, England), 1,000 mg/L, in 2% HNO_3_, standard of the Ca, Cr, Ni, Cu, Zn, Cd and Pb (BDH Chemicals Ltd Spectrosol^®^, Poole, England) and H_2_O_2_. Deionized water was used for dilution of sample and intermediate metal standard solutions prior to analysis and rinsing glassware.

### Sample collection

All the cannabis samples analyzed in this investigation were confiscated during 2013. Three of the samples were whole fresh plants collected from the growing plots by the police before they had been harvested except samples from Mekelle which was collected from the users. Leaves of cannabis samples caught by police were collected from the four sites. The four sites are Butajira (SNNP Region), Metema (Amhara Region), Sheshemene (Oromia Region) and Mekelle (Tigray Region). About 200 g of fresh leaves of cannabis was taken from each sites except Mekelle (Tigray Region) and the collected samples were packed into polyethylene plastic bags, labeled and transported to the laboratory for further treatment. The geographical locations of these sampling sites are described in Table 1.

**Table 1:** Geographical description of sample collection sites

No.	Sample sites	Approximate geographical locations			
		Latitude	Longitude	Altitude in meters (above sea level)	Distance in kilometers and directions from Addis Ababa
1	Butajira (SNNP Region)	8°10′N	38°50′E	1,900	132 km South West
2	Metema (Amhara Region)	12°58′N	36°12′E	684	898 km North West
3	Sheshemene (Oromia Region)	7°12′N	38°36′E	2,009	240 km South East
4	Mekelle (Tigray Region)	13°29′N	39°28′E	2,037	787 km North

### Sample preparation for elemental analysis

The cannabis samples were washed with a running tap water so as to remove adsorbed soil particulates and then rinsed with deionized water. The samples were exposed to sun light until the weight becomes constant for several days to reduce the moisture content so as to express the result in terms of dry mass basis. The dried sample was ground using electronic blender and sieved to prepare fine powder of cannabis for digestion. Then the fine powder of cannabis samples were kept in properly washed, dried and cleaned polyethylene plastic bags until appropriate amounts of the samples were taken for digestion.

### Optimization of digestion procedure

The basic requirements for sample preparation for analysis are to get an optimum condition for digestion. The optimum condition is the one which required minimum reagent volume consumption, minimum reflux time, clarity of digests, and ease of simplicity (Soylak et al. 2004; Tuzen and Soylak 2007; Uluozlu et al. 2007; Demirel et al. 2008).

In wet acid digestion the optimized digestion procedure is attained based on changing different digestion parameters like volume ratio of reagents added, digestion temperature and digestion time. In this procedure the organic components are decomposed in the different gaseous forms and other metallic elements are left in the solution except those volatile metals like Hg. The digestion is assumed to be complete if the solution is clear and colorless (Endalamaw and Chandravanshi 2015; Wagesho and Chandravanshi 2015). Based on this fact different digestion procedures were optimized using the HNO_3_:HCl:H_2_O_2_:HClO_4_ acid mixtures by varying parameters such as volume of the acid mixture, digestion time and digestion temperature. The results of optimization procedures for the analysis of *Cannabissativa* L. samples for its metals content are shown in Tables 2, 3, 4.

**Table 2:** Optimization of volume ratio of the reagents for digestion of sample mass (0.5 g) at 300°C for 4 h

No.	Total volume (mL)	Volume ratio (mL) HNO_3_: HCl:H_2_O_2_:HClO_4_	Observation
1	4	2:0:1:1	Pale yellowish solution with small residue
2	4	2:1:0:1	Yellowish solution with residue
3	4	2:0.5:0.5:1	Pale yellow solution with residue
4	5	2:2:0:1	Pale yellow solution with residue
5	5	2:1:1:1	Yellow solution with residue
6	5	3:1:1:0	Deep yellow solution with small residue
7	6	3:1:1:1	Yellow solution with small residue
8	6	3:2:0.5:0.5	Colorless solution but not clear
9	7	4:1:1:1	Pale yellow solution with small suspension
10	7	4:2:0.5:0.5	Yellow solution with residue
11	8	5:1:1:1	Slightly clear and colorless solution with residue
12	8	5:2:0.5:0.5	Pale yellow solution with small suspension
13	9	6:2:0:1	Slightly clear and colorless solution with residue
14	9	7:0:1:1	Slightly clear and colorless solution with small residue
15	*10*	*9:0:0:1*	*Clear and colorless solution*
16	10	9:0.5:0:0.5	Clear and colorless solution
17	10	9:0:0.5:0.5	Clear and colorless solution

Italics indicate optimum volume ratio of reagents for digestion.

**Table 3:** Optimization of digestion temperature for digestion of sample mass (0.5 g) for volume (10 mL) for 4 h

No.	Temperature (^o^C)	Observation
1	180	Slightly colorless solution with residue
2	210	Slightly colorless solution with residue
3	240	Slightly colorless solution with residue
4	*300*	*Clear and colorless solution*

Italics indicate optimum digestion temperature.

**Table 4:** Optimization of digestion time for digestion of sample mass (0.5 g) for volume (10 mL) at 300°C

No.	Time (h)	Observation
1	2:00	Colorless solution with some residue
2	2:30	Colorless solution with some residue
3	3:30	Clear and colorless with some residue
4	*4:00*	*Clear and colorless*

Italics indicate optimum digestion time.

### Digestion of leaves of *Cannabis sativa* L. samples

Applying the optimized conditions (Tables 2, 3, 4), 0.5 g of dried and homogenized cannabis samples were transferred into a 250 mL round bottom flask. Then 10 mL of a mixture of HNO_3_ (69.5%) and HClO_4_ (70%) with a volume ratio of 9:1 (v/v) was added and the mixture was digested on a Kjeldahl digestion apparatus fitting the flask to a reflux condenser by setting the temperature at 300°C for 4:00 h. The digest was allowed to cool to room temperature for 30 min without dismantling the condenser from the flask and for 10 min after removing the condenser. To the cooled solution 10 mL of deionized water was added to dissolve the precipitate formed on cooling and to minimize dissolution of filter paper by the digest residue while filtering with Whatmann (110 mm diameter) filter paper into 25 mL volumetric flask. The round bottom flask was rinsed subsequently with 5 mL deionized water until the total volume reached around 15 mL. To this final solution, about 0.67 g of La(NO_3_)_3_.6H_2_O was added to prevent the precipitation of Ca^+2^ with the SO_4_^−2^ and PO_4_^−3^ and the solution was filled to the mark (25 mL) with deionized water. The digestion was carried out in triplicate for each bulk sample. Digestion of a reagent blank was also performed in parallel with the cannabis samples keeping all the digestion parameters the same. The digested samples were kept in the refrigerator, until the level of all the metals in the sample solutions were determined by FAAS.

### Instrument calibration

Metal standard solutions for calibration were prepared for each of the metals from an intermediate standard solution containing 10 mg/L which was prepared from the atomic absorption spectroscopy standard stock solutions containing 1,000 mg/L. These intermediate standards were diluted with deionized water to obtain four working standards for each metal of interest. The absorbances of the working standard solutions were measured and the calibration curves for each of the analyte metal (Ca, Cr, Ni, Cu, Zn, Cd and Pb) were constructed. The levels of each metal in all the samples were determined with FAAS using external calibration curve after the instrument parameters were optimized for maximum signal intensity. Three replicate determinations were carried out on each samples and standards. The same analytical procedure was employed for the determination of elements in digested blank solutions.

Concentration of working standards, correlation coefficient of the calibration curves and equation for calibration curves for each metal are listed in Table 5. The correlation coefficients in Table 5 for each metals show that the change in absorbance with concentration is in good positive correlation and linear fit.

**Table 5:** Wavelength, working standard concentration, correlation coefficient and equation of the calibration curves for determination of metals using FAAS

Metal	Wavelength (nm)	Method detection limit (μg/g dry weight)	Concentration of working standards (mg/L)	Correlation coefficient (r)	Equation for calibration curves
Ca	422.7	25	0.25, 0.5, 0.75, 1.0	0.9997	Y = 0.49265 X − 2.64 × 10^−3^
Cr	357.9	1	1.0, 2.0, 3.0,4.0	0.9997	Y = 0.09698 X + 5.00 × 10^−4^
Ni	232.0	8	1.0, 2.0, 3.0,4.0	0.9998	Y = 0.04318 X − 6.22 × 10^−3^
Cu	324.8	19	0.25, 0.5, 1.0, 2.0	0.9999	Y = 0.20258 X − 2.80 × 10^−4^
Zn	213.9	31	0.25, 0.5, 0.75, 1	0.9999	Y = 0.66746 X − 1.13 × 10^−3^
Cd	228.8	0.4	0.25, 0.5, 0.75, 1	0.9997	Y = 0.49265 X − 2.64 × 10^−3^
Pb	217.0	6.0	1.0, 2.0, 3.0,4.0	0.9995	Y = 0.09786 X + 4.52 × 10^−3^

### Method detection limits (MDL)

The method detection limit is the minimum concentration that can be detected by the analytical method with a given certainty. It is also the smallest concentration or amount of an analyte that can be reliably shown to be present or measured under defined condition, and the limit of detection is the lowest concentration level that can be determined statistically different from a blank (99% confidence). The MDL/LOD is typically determined to be in the region where the signal-to-noise ratio is greater than 3 but not necessarily quantified as an exact value (Chen 2007). It can be calculated by multiplying the pooled standard deviation of the reagent blank (S_blank_) by three (MDL = 3 × S_blank_, n = 12). Method detection limits of the metals of interest are shown in Table 5 which indicates that the presence of trace amounts of metals of interest in the sample can be detected by the method.

### Validation of optimized procedure

The validity of the optimized procedure was assessed by spiking experiments. For this purpose standard solution of 1,000 mg/L was used and intermediate standards of 10 mg/L were prepared. For recovery measurement Amhara sample was selected randomly. The spiking was done in three groups in triplicate.

In the first group 1.4 µL of 1,000 mg/L of Ca and 240 µL of 10 mg/L of Zn were spiked. In the second group 12.2 µL of 1,000 mg/L of Cu, 40 µL of 10 mg/L of Cr and 7.4 µL of 1,000 mg/L of Ni were spiked. In the third group 210 µL of 10 mg/L of Pb and 80 µL of 10 mg/L of Cd were spiked.

The spiked, un-spiked and reagent blank samples were digested and analyzed in similar condition using the optimized procedure as used for sample analysis. The results of recovery analysis are shown in Table 6 and the percentage recoveries lies within the range 88–103%. The percentage recovery for cannabis samples are within the acceptable range for all the metals.

**Table 6:** Recovery test using optimized procedure of metals in leaves of *Cannabis sativa* L. sample

Metal	Amount spiked (mg in 0.5 g)	Amount recovered (mg in 0.5 g)	Percent recovery (% R)
Ca	0.0014	0.0013	92.9
Cr	0.0004	0.0004	100
Ni	0.0074	0.0072	97.3
Cu	0.0122	0.0125	102.5
Zn	0.0024	0.0022	91.7
Cd	0.0008	0.0007	87.5
Pb	0.0021	0.0019	90.5

## Results and discussions

### Levels of metals in leaves of *Cannabis sativa* L. samples

The developed FAAS method was applied for the determination of the levels of seven metals (Ca, Cd, Cr, Cu, Ni, Pb, Zn) in leaves of *Cannabis sativa* L. samples collected from four different sites of Ethiopia, i.e. Butajira (SNNP Region), Metema (Amhara Region), Sheshemene (Oromia Region) and Mekelle (Tigray Region). From statistical analysis of percent relative standard deviation (% RSD), all the determined concentrations are within the range of mean ± 15%. The mean values were determined from triplicate analysis of each sample. Results obtained for each sample in terms of μg/g dry weight basis together with values for standard deviation are shown in Table 7.

**Table 7:** Mean concentration (mean ± SD, n = 9, μg/g dry weight) and standard deviation of selected metals in each sample analyzed by FAAS

Element	Sample sites			
	Butajira (SNNP Region)	Metema (Amhara Region)	Sheshemene (Oromia Region)	Mekelle (Tigray Region)
	Mean ± SD	Mean ± SD	Mean ± SD	Mean ± SD
Ca	868 ± 71	1,511 ± 53	657 ± 76	1,210 ± 130
Zn	377 ± 20	315 ± 9.8	380 ± 43	321 ± 15
Ni	149 ± 19	124 ± 13	162 ± 14	172 ± 18
Cu	122 ± 14	141 ± 16	176 ± 23	165 ± 16
Cd	3.2 ± 0.2	3.7 ± 0.4	4.7 ± 0.3	3.4 ± 0.2
Pb	8.3 ± 0.9	10.2 ± 1.6	7.9 ± 0.3	9.6 ± 0.7
Cr	3.9 ± 0.6	7.6 ± 1.3	3.6 ± 0.2	3.8 ± 0.3

### Distribution patterns of metals in the samples

Metals uptake by plants may occur through different and complex biochemical processes. This uptake varies based on the ability of the plants to absorb metals from the soil, the availability of the mineral elements in soluble and absorbable forms, the abundance of specific metals at the specified site, the contamination level of the soil with heavy metals, etc. The variation of metal levels in soil arises because of increasing industrialization and associated pollution of the biosphere, use of different types of fertilizers, pesticide treatment, and others are the main contributors. The use of sewage sludge, pesticides, irrigation of waters and fertilizers on agricultural land has made some of that land of questionable quality for production of food for humans and animals. The distribution and accumulation of metals in leaves of *Cannabis sativa* L. are the reflection of the mineral composition of the soil and environment in which *Cannabis sativa* L. plant grows. Therefore, the actual metal content of cannabis vary considerably according to geographic origin, the use of fertilizers with different chemical compositions and other characterizing features such as water used for irrigation.

Plants accumulate metals from the soil and environment in its different parts. This study focuses the level of metals in leaves of cannabis because it is the leaves that are commonly consumed by users. There is variation in Ca concentration among the determined samples. Statistical evaluation using one-way ANOVA revealed that there is significant difference in Ca content among the four sample sites at the 95% confidence level. It is important to realize that the one-way ANOVA does not tell which specific sample sites are significantly different from each other; it only tells that at least two groups are different. Since we have four groups in the study, determining which of these groups differ from each other is important. For this, multiple comparisons among the Ca concentrations of the four samples was performed by using the Tukey post hoc test. The results indicated that both the cannabis samples from Mekelle (Tigray Region) and Metema (Amhara Region) sites differ significantly from each other as well as from Sheshemene (Oromia Region) and Butajira (SNNP Region) samples. On the other hand, there is no significant difference in Ca contents between samples from Sheshemene (Oromia Region) and Butajira (SNNP Region) sites. Accordingly, three homogenous subsets of sample sites are identified based on their mean calcium concentrations: Metema (Amhara Region) (1,511 μg/g) > Mekelle (Tigray Region) (1,210 μg/g) > Butajira (SNNP Region) (868 μg/g) and Sheshemene (Oromia Region) (657 μg/g).

Zn (315–380 μg/g dry weight) was the most accumulated trace metal followed by Cu (122–176 μg/g dry weight) and Ni (124–172 μg/g dry weight) in the cannabis sample. However, the determined concentrations of Cu and Ni overlap each other among the sample regions, while the value of Zn is higher in all samples as compared to that of Cu and Ni. The higher concentration of Zn might be attributed to its relative abundance in the earth’s crust relative to copper.

The highest concentration of Zn was determined in samples from Sheshemene (Oromia Region) (380 ± 43 μg/g dry weight) followed by Butajira (SNNP Region) (377 ± 20 μg/g dry weight) and lowest in samples from Metema (Amhara Region) (315 ± 9.8 μg/g dry weight).

The trend of concentration of trace metals in cannabis samples collected from each of the sample sites was as follows: both Butajira (SNNP Region) and Mekelle (Tigray Region): Zn > Ni > Cu; both Metema (Amhara Region) and Sheshemene (Oromia Region): Zn > Cu > Ni. The individual metal concentrations by sample region indicates that the trend for Zn decreased with the order Sheshemene (Oromia Region) ≈ Butajira (SNNP Region) > Mekelle (Tigray Region) ≈ Metema (Amhara Region). For Cu the trend was Sheshemene (Oromia Region) > Mekelle (Tigray Region) > Metema (Amhara Region) > Butajira (SNNP Region). The trend for Ni was Mekelle (Tigray Region) > Sheshemene (Oromia Region) > Butajira (SNNP Region) > Metema (Amhara Region).

The results obtained for Zn in cannabis indicate that the variation by sample sites is very narrow. Further statistical treatment of data with one-way ANOVA suggested that significant difference exists in the determined concentration of Zn among the four sample sites at the 95% confidence level.

The result from one-way ANOVA with the level of Cu in cannabis samples indicates that the variation of Cu by sample site is significant at the 95% confidence level. Multiple comparison among the mean Cu concentrations shows that significant difference exists specifically only between samples from Butajira (SNNP Region) and Sheshemene (Oromia Region). On the other hand, there is no significant difference in the levels of Cu found in Mekelle (Tigray Region) and Metema (Amhara Region) samples when compared with each other as well as with samples from Butajira (SNNP Region) and Sheshemene (Oromia Region).

Similarly, the result from one-way ANOVA with the level of Ni in cannabis samples indicates that the variation of Ni by sample site is significant at the 95% confidence level. Multiple comparisons among the mean Ni concentrations show exactly the opposite of the variation observed for the levels of Cu in the four sample site types. Significant difference exists specifically between samples from Metema (Amhara Region) and Mekelle (Tigray Region). Contrary to the trend observed for copper, there is no significant difference in the levels of Ni found in Butajira (SNNP Region) and Sheshemene (Oromia Region) samples when compared with each other as well as with samples from Mekelle (Tigray Region) and Metema (Amhara Region).

In this study, cannabis samples were also analyzed for the contents of three toxic metals Pb, Cr and Cd. The concentration of Pb determined in all of the samples is found to be higher than the other toxic metals determined in this study, i.e. Cd and Cr. Furthermore, there is no statistically significant difference in the level of Pb among the four sample sites at 95% confidence level.

It should be noted that Cr(III) is essential but Cr(VI) is non-essential to human. In this study the total Cr was determined and therefore it was not possible to know the specific form in which Cr exist in the leaves of *Cannabis sativa* L.

The amount of Cr determined in the samples from Metema (Amhara Region) is statistically significantly higher than those from the other sites. Whereas, the amount of Cr found in samples from Mekelle (Tigray Region), Sheshemene (Oromia Region) and Butajira (SNNP Region) appear to be statistically similar. Consequently, two statistical homogeneous subsets are identified with one comprising samples from Mekelle (Tigray Region), Sheshemene (Oromia Region) and Butajira (SNNP Region) while samples from Metema (Amhara Region) representing the other subset. On the other hand, the sample obtained from Sheshemene (Oromia Region) is found to contain statistically significantly higher Cd than those obtained from the other sites. Whereas, the amount of Cd found in samples from Mekelle (Tigray Region), Metema (Amhara Region) and Butajira (SNNP Region) appear to be statistically similar. Accordingly, two statistical homogeneous subsets are identified with one consisting of samples from Mekelle (Tigray Region), Metema (Amhara Region) and Butajira (SNNP Region) while samples from Sheshemene (Oromia Region) representing the other subset.

### Comparison of metal levels in leaves of *Cannabis sativa* L. obtained in the present study with literature values

The comparative levels of metals in cannabis found in this study with the levels reported in the literatures are shown in Table 8. The results in the Table 8 revealed that the levels of metals determined in this work are in good agreement with other researchers’ work except for Ni and Zn.

**Table 8:** Comparison of selected metal concentrations (µg/g, dry weight basis) in leaves of cannabis samples with reported values

Concentration of metal (mg/kg)							Country	References
Ca	Ni	Cr	Cu	Zn	Cd	Pb		
2,100	NR	NR	NR	54.0	3.7	NR	Not mentioned	Mihoc et al. (2012)
NR	10.4	17.4	NR	NR	4.4	1.6	Nigeria	Eboh and Thomas ( 2005)
NR	3.3–3.6	3.9–4.0	NR	NR	0.03–0.07	6.1–6.7	Pakistan	Khan et al. (2013)
NR	1.2	0.1	0.7	0.2	0.02	0.36	Pakistan	Zehra et al. (2009)
NR	NR	NR	1,330	112.5	NR	15	Pakistan	Ghani et al. (2012)
1,425.9	94	205.2	388	1,700.5	BDL	BDL	India	Tiwari et al. (2014)
657–1,511	124–172	3.6–7.6	122–176	315–380	3.2–4.7	7.9–10.2	Ethiopia	This study

The methods used in all the references were AAS.

*NR* not reported, *BDL* below detection limit.

As it is shown in Table 8, the concentration of Ca (657**–**1,511 μg/g dry weight) determined in this study is in good agreement with Tiwari et al. (2014) (1,426 μg/g dry weight). However, it is less than that reported by Mihoc et al. (2012) (2,100 μg/g dry weight). The result in this work and also in the reported work showed that Ca is the one with highest concentration among all investigated metals.

The Zn content of cannabis determined in this study (315**–**380 μg/g dry weight) is in the range reported by Zehra et al. (2009) (0.2 μg/g dry weight) and Tiwari et al. (2014) (1,701 μg/g dry weight). However, with all other reports in Table 8 the present data is not comparable. Likewise, the Ni content of this study is not comparable with reported values. But, to the contrary of Zn, in all cases the concentration of Ni in this study (124–172 μg/g dry weight) is higher than reported by Eboh and Thomas (2005) (10.4 μg/g dry weight), Khan et al. (2013) (3.3–3.6 μg/g dry weight), Zehra et al. (2009) (1.2 μg/g dry weight) and Tiwari et al. (2014) (94 μg/g dry weight). The concentration of Cu in this study (122**–**176 μg/g dry weight) is in the range between the values reported by Zehra et al. (2009) (0.7 μg/g dry weight) and Tiwari et al. (2014) (388 μg/g dry weight). However, it is too lower and higher than the levels reported by Ghani et al. (2012) (1,330 μg/g dry weight) and Zehra et al. (2009) (0.7 μg/g dry weight), respectively. In general the concentrations of all micro-essential metals are in the range of the reports, except Ni.

The level of Cr in this study (3.6–7.6 μg/g dry weight) is comparable to that reported by Khan et al. (2013) (3.9–4.0 μg/g dry weight), but much lower than those determined by Tiwari et al. (2014) (205 μg/g dry weight) and Eboh and Thomas (2005) (17.4 μg/g dry weight). To the contrary it is much higher than that was determined by Zehra et al. (2009) (0.1 g/g dry weight).

In this study the level of Cd (3.2–4.7 μg/g dry weight) is comparable with the levels reported by Mihoc et al. (2012) (3.7 μg/g dry weight) and Eboh and Thomas (2005) (4.4 μg/g dry weight) but too higher than the levels reported by Khan et al. (2013) (0.03–0.07 μg/g dry weight) and Zehra et al. (2009) (0.2 μg/g dry weight). The level of Pb (7.9–10.2 μg/g dry weight) is comparable with the levels reported by Khan et al. (2013) (6.1–6.7 μg/g dry weight) and Ghani et al. (2012) (15 μg/g dry weight). However, it is much higher than the values reported by Eboh and Thomas (2005) (1.6 μg/g dry weight) and Zehra et al. (2009) (0.36 μg/g dry weight). Overall, all metals which were investigated in this study are in the range of the reported values except Ni.

### Statistical analysis

In analytical work two or more mean results are compared to check whether there is a significant difference between them or not. The variation between the means of different samples can result from random and controlled sources of error. This kind of variation and the variation due to the difference in the original sample type can be separated and estimated by a powerful statistical technique known as analysis of variance (ANOVA) (Miller and Miller 2005). In this study, a one way ANOVA and Pearson correlation coefficient was used to decide the variation between samples analyzed was significant or not for the mean concentrations of each metal in triplicate analysis.

### Analysis of variance

The variation in samples means come from different sources such as experimental procedure or heterogeneity among the samples (i.e. difference in mineral contents of soil, pH of soil, water, atmosphere; variation in application of agrochemicals like fertilizers, pesticides, herbicides, etc. or other variations in cultivation procedures). In this study the variation in samples means of the analyte was tested whether they have significant difference or not by using ANOVA of SPSS 20 (IBM) software (Table 9).

**Table 9:** Analysis of variance (ANOVA) between and within cannabis samples at 95% confidence level

	df	F	Significance
Ca			
Between samples	3	55	0.000
Within samples	8		
Total	11		
Zn			
Between samples	3	6	0.022
Within samples	8		
Total	11		
Ni			
Between samples	3	5	0.034
Within samples	8		
Total	11		
Cu			
Between samples	3	6	0.022
Within samples	8		
Total	11		
Cd			
Between samples	3	19	0.001
Within samples	8		
Total	11		
Pb			
Between samples	3	4	0.068
Within samples	8		
Total	11		
Cr			
Between samples	3	19	0.001
Within samples	8		
Total	11		

From Table 9 one can see that there is significant difference at F_3,8_ at 95% confidence level in mean concentration for all metals except Pb. This is due to variations in the other factors than in the experimental procedure. The source for this significant difference between sample means may be the difference in mineral contents of soil or pH of soil which predict the extent of mineral absorption by cannabis.

### Pearson correlation of metals within cannabis samples

In this study, to correlate the effect of one metal concentration on the concentration of the other metal, the Pearson correlation matrices using correlation coefficient (r) for the samples were used and presented in Table 10. The data in Table 10 indicate that most of the metals have negative correlation with each other except Ca having positive correlation with Cr and Pb; Zn with Ni, Cu, Cd; Ni with Cu; Cu with Cd; and Pb with Cr.

**Table 10:** Pearson correlation matrices for metals in leaves of *Cannabis sativa* L. samples (n = 12)

	Ca	Zn	Ni	Cu	Cd	Pb	Cr
Ca	1						
Zn	−0.797**	1					
Ni	−0.508	0.305	1				
Cu	−0.187	0.193	0.313	1			
Cd	−0.383	0.251	−0.029	0.568	1		
Pb	0.698*	−0.637*	−0.083	−0.268	−0.310	1	
Cr	0.766**	−0.543	−0.675*	−0.309	−0.098	0.703*	1

* Correlation is significant at the 0.05 level (2-tailed).

** Correlation is significant at the 0.01 level (2-tailed).

## Conclusion

In this study the levels of metal in *Cannabis sativa* L. collected from four regions of Ethiopia were analyzed for their contents of Ca, Cr, Ni, Cu, Zn, Cd and Pb using flame atomic absorption spectrometer. The optimized wet digestion method for the analysis of cannabis was found efficient for all the metals and it was evaluated through the recovery experiment and a good percentage recovery of 88–103% was obtained for all the metals identified.

The level of metals investigated in cannabis is in the following order: Ca (657–1,511 μg/g) > Zn (315–380 μg/g) > Ni (124–172 μg/g) > Cu (122–176 μg/g) > Pb (7.9–10.2 μg/g) > Cr (3.6–7.6 μg/g) > Cd (3.2–4.7 μg/g).

Statistical analysis by using one way ANOVA indicates that there is significant difference in mean concentration of metals in *Cannabis sativa* L. in the four sampling sites except Pb. This may be attributed to differences in soil composition, use of different fertilizers, pesticides, etc. and for Pb, the difference may only be attributed to random errors in the experimental procedures. Pearson correlation matrices indicate that most of the metals have negative correlation with each other except Ca having positive correlation with Cr and Pb; Zn with Ni, Cu, Cd; Ni with Cu; Cu with Cd; and Pb with Cr. This clearly indicates that most metals have different sources of accumulation in *Cannabis sativa* L.

Based on the results of this study, it can be concluded that *Cannabis sativa* L. contains higher levels of essential metals Ca, Zn and Cu. But highly toxic metals like Pb and Cd were also detected in *Cannabis sativa* L. The content of both toxic metals are beyond permitted limits set by WHO. This will further lead to the harmful effect of *Cannabis sativa* L. on human health. It is necessary to monitor the levels of these toxic metals in medicinal herbs.

In general, the levels of metals in the Ethiopian cannabis are comparable to those reported in the literature from other countries.
